# Solitary fibrous tumor of central nervous system masquerading as meninigioma: Report of a rare case

**DOI:** 10.1016/j.ijscr.2018.11.063

**Published:** 2018-11-27

**Authors:** Sant Prakash Kataria, Namita Bhutani, Sanjay Kumar, Gajender Singh, Rajeev Sen, Ishwar Singh

**Affiliations:** Deptt. of Pathology, PGIMS Rohtak, Haryana, India

**Keywords:** Central nervous system, Hemangiopericytoma, Intracranial, Solitary fibrous tumor

## Abstract

**Introduction:**

Solitary fibrous tumor (SFT) is a rare neoplasm of mesenchymal origin that occurs most often in the visceral pleura. It rarely occurs in the central nervous system (CNS). If they occur intracranially, they are extra-axially located and develop from the meninges.

**Case Report:**

Herein, we present a case of SFT in a 45 years old female. MR imaging showed isointense to hypointense signal intensity, and marked enhancement. Microscopically, the tumors showed characteristic “patternless-pattern” architecture. Elongated tumor cells formed fascicles alternating with hypocellular densely collagenous stroma. Immunohistochemistry for STAT6, CD34, BCL2 and CD99 favours the definitive diagnosis of SFT.

**Conclusion:**

In general, complete surgical resection may offer the best chance of a favourable clinical outcome. Their biological features remain largely unknown. Hence, the clinical management and prognosis is often challenging due to the lack of comprehensive data.

## Highlights

• Solitary fibrous tumors (SFTs) are uncommon spindle cell neoplasms of mesenchymal origin.
• Involvement of the CNS is extremly rare and has been attributed to the paucity of true connective tissue elements.
• It is reported that central nervous system SFTs account for ∼0.09% of meningeal tumors.
• These tumors are considered to be benign at the onset although anaplastic or malignant transformation has also been reported.

## 1 Introduction

Solitary fibrous tumors (SFTs) are rare spindle cell neoplasms of mesenchymal origin. These were initially described as primary neoplasms of the mediastinum and visceral pleura by Klempere and Rabin in 1931 [1]. However, they have also been reported under various other labels in a large number of extrathoracic body sites, including head and neck, pericardium, peritoneum, liver, thyroid, mesentery, as well as the sinus and orbit but only rarely in the central nervous [2]. Primary SFT involving the central nervous system (CNS) was first reported in 1996 by Carneiro et al [3]. Involvement of the CNS is extremely rare and has been attributed to the paucity of true connective tissue elements. It is reported that central nervous system SFTs account for ∼0.09% of meningeal tumors [4]. Over the past 10 years, a few studies on central nervous system SFTs have been reported, with the majority of cases located in the spinal cord. Intracranial lesions, however, are not always observed. When they do occur intracranially, they are usually extra-axially located.

CNS SFT has been reported at the cerebellopontine angle, spinal dura, parasagittal region, meninges, and the intraventricular region [5]. It might involve the nerve roots as well as the skull base. To date, the clinical course, histogenesis, cytogenetics and prognosis for intracranial SFTs remain largely debatable. The tumor entity was carefully reexamined and further characterized as fascicles of spindle cells, resembling CD34-positive interstitial dendritic cells intermingled with bands of collagen. Pre-operative definitive diagnosis is difficult due to the atypical symptoms and imaging characteristics and therefore, it mainly depends on the postoperative pathological examination. These tumors were considered to have benign histopathological features at onset although anaplastic or malignant transformation resulting from multiple recurrences has also been reported [6]. SFT is thought to arise from the fibroblast and needs to be differentiated from some tumors, like fibrous meningioma and hemangiopericytoma, and from myxoid variants like myxochordoid meningioma and myxoid peripheral nerve sheath tumor [7]. The fact that this entity is clinically distinct from other mesenchymal extracranial soft tissue tumors, often leading to an unusual clinicopathological presentation and outcome pattern, different from other benign and malignant spindle cell tumor entities remains largely unknown to most neurosurgeons [6]. Reported herein is a case of CNS SFT in a 45-year-old female patient.

## 2 Case report

**Fig. 1:**
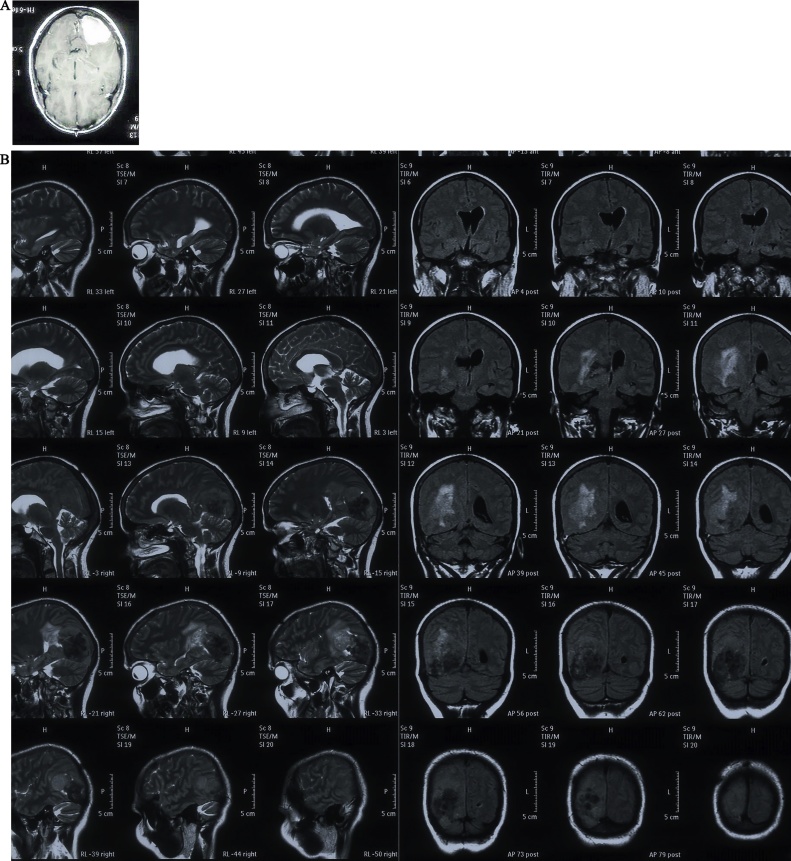
A: On MRI an ovoid mass lesion seen in the right parieto-occipital region measuring 10 × 7 × 4 cm with peritumoral edema. B: The tumor showed intermediate-low signal intensity in the T1-Weighted Image (T1WI). The mass was attached to tentorium and was seen extending into the right transverse sinus.

**Fig. 2:**
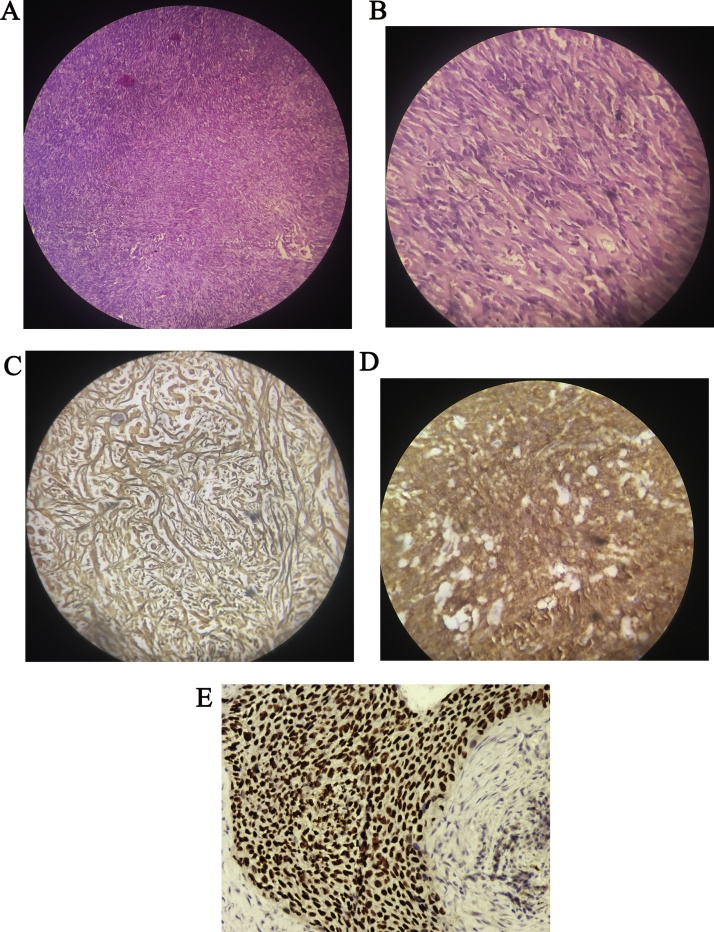
A: Photomicrograph depicting a spindle cell tumor with a “PAtternless-pattern with hypercellular and hypocellular areas. (H& E-40 X). B: Tumor cells are spindle shaped and collagen fibres are seen to separate them. Hypercellular areas showed interlacing fascicles of spindle-shaped cells with moderate amount of eosinophilic cytoplasm and oval to elongated nuclei exhibiting variable pleomorphism. (H&E-200X). C: Reticulin fibres are seen to separate the tumor cells (RETICULIN-200X). D: On IHC, CD 99 Is strongly positive in tumor cells. (100×). E: Stat 6 strongly positive in tumor cells (100X).

A 45 years old female presented to neurology outpatient department with complains of headache and dizziness for 1 month. Her neurologic examination was normal. The electro encephalogram (EEG) showed deceleration in the right hemisphere, but no other abnormalities. The brain magnetic resonance imaging (MRI) showed a 10 × 7 × 4 cm ovoid mass in the right parieto-occipital region with peritumoral edema. The mass was attached to tentorium and was seen extending into the right transverse sinus. The tumor showed intermediate-low signal intensity in the T1-weighted image (T1WI) and slightly increased signal intensity in T2-weighted image (T2WI). The upper and medial portions of the mass showed heterogeneous and relatively low signal intensity in T2WI and suggested a fibrotic mass. The mass showed strong enhancement in the gadolinium-enhanced T1 image. However, we felt that there was also the possibility that the lesion was dura based and simply compressing the ventricle. Given the imaging characteristics, a provisional diagnosis of a meningioma was made (Fig. 1A and B). Gross total resection was done. The tumor was a well-encapsulated, greyish white solid, round and firm mass. The pathologic examination revealed a spindle cell tumor with a “patternless-pattern”. The tumor showed variable cellular morphology comprising of mixed hypercellular and hypocellular areas, with multifocal intervening collagen lay down and scattered vessels. Hypercellular areas showed interlacing fascicles of spindle-shaped cells with moderate amount of eosinophilic cytoplasm and oval to elongated nuclei exhibiting variable pleomorphism. Hypocellular areas showed spindle cells with bland nuclear chromatin and abundance of collagen. The tumor cells showed diffuse, strong immunoreactivity for STAT 6, CD 99, CD34, BCL-2 and Vimentin. The mitosis was less than 1/10 high power field (HPF), with an about 1% Ki-67 labelling index, and there was no evidence of necrosis. Sparse reticulin fibers were observed amongst the tumor cells on special stain. With these results, hemangiopericytoma was ruled out, and a diagnosis of SFT was made (Fig. 2A–D). The post-operative neurological status was substantially improved and regular follow-up examinations for 6 months post-surgery have shown that the patient is currently disease-free. The patient was scheduled for follow-up MRI after three months.

## 3 Discussion

Solitary fibrous tumours are rare spindle-cell mesenchymal tumours that mostly occur in the visceral pleura but have been reported to occur outside the thoracic cavity also. The sites of involvement include the pericardium, peritoneum, lung, liver, upper respiratory tract, mediastinum, thyroid glands, and parotid glands. Central nervous system SFTs account for only 0.09% of all meningeal tumors, while intracranial SFTs (ISFT) are extremely rare [6]. The most frequent location is along the tentorium cerebelli, followed by the frontal convexity, cerebellopontine angle, ventricles, falx cerebri, and posterior fossa [3]. There has been debate over the histogenesis of the SFT as to whether the origin is a mesothelial or mesenchymal, but recent immunohistochemical and electron microscopic studies have suggested that they originate from mesenchymal fibroblast-like cells.

The majority of intracranial SFTs occur in the fifth decade and are seen in females. The tumors grow slowly and patients may present with several non-specific symptoms related to increased intracranial pressure or with the location of the tumor. Only when the lesions become large enough or infringe into the important functional areas clinical symptoms arise. The symptoms include episodic headaches, gait imbalance, dizziness, sensory disturbance, hemiplegic paralysis and epileptic seizure [8].

Radiologically, it is difficult to differentially diagnose SFT on imaging.The most common presentation is a heterogeneous pattern on T2-weighted imaging with hypointense areas. This may be explained by the existence of two separate components within the tumour. The first component is fibrosis, represented by T2-hypointense areas that show intense contrast enhancement. The second hypercellular component presents as a T2 iso- or hyperintense area with moderate heterogeneous enhancement. The combination of these two components gives rise to the so-called yin yang sign, which is associated with ISFTs. SFTs are frequently misdiagnosed as other tumors, including neurofibroma, fibrous meningioma, hemangiopericytoma, malignant fibrous histiocytoma and fibrosarcoma, on MRI. An intracranial solitary fibrous tumour and a hemangiopericytoma are both compatible with the imaging characteristics. These tumours cannot be distinguished from one another through imaging. They therefore require diligent work-up to distinguish amongst these rare but distinct CNS tumors and possible differentials [8].

The characteristic histologic features of SFT include the so-called “patternless-pattern” of spindle cells, hemangiopericytoma-like pattern of vascularity, and thick strands of stromal collagen. Alternating hypo- and hypercellular areas is another important clue on low-power examination. A very close differential is hemangiopericytoma. But hemangiopericytomas (HPCs) are remarkable for their characteristic thin-walled, branching or “staghorn” blood vessels. Although cellularity is variable, HPCs are often composed of closely packed, randomly oriented cells with irregular, carrot-shaped nuclei. The classic histologic features combined with immunohistochemistry are helpful in reaching to a correct diagnosis. Immunohistochemically, SFTs are in most cases diffusely positive for STAT 6 CD34, while meningiomas are typically reactive for EMA. However, CD34 is not specific for SFTs, as weak and usually patchy staining may be visualized in meningiomas, neurofibromas, and hemangiopericytomas. Positivity for CD99 and bcl-2 is found in more than half of all cases of SFTs and they also stain strongly with the intermediate filament vimentin, but are usually negative for the neural crest markers S-100, GFAP, EMA, cytokeratin, or vascular antigens [3]. However, Corinne et al found overlapping pathological features and common prognostic factors between SFT and HPC, suggesting that they belong to the same spectrum of tumors [3]. Electron microscopy has not yielded a unique distinctive feature, but some typical aspects include a well-developed rough endoplasmatic reticulum, occasional primitive junctions, and a lack of desmosomes as well as basal lamina. For these reasons, immunohistochemistry is essential in making the primary diagnosis, when a complex differential diagnosis is entertained.

Surgery offers the best first-line treatment and achieves excellent local control.Total excision of the mass is clearly superior to subtotal resection, with a 16-fold increase in recurrence ratio with subtotal resection (without adjuvant therapy), compared to total resection. Conventional radiotherapy as well as stereotactic radiosurgery (SRS) has been described in occasional reports as adjuvant treatment for residual SFTs. However, the role of adjuvant postoperative radiotherapy in improving long-term prognosis remains unclear and the number of patients who underwent complementary chemotherapy treatment is too small to evaluate any possible benefit. Gamma Knife radiosurgery (GKRS) is a feasible adjunct for treating SFT; however, we still recommend close and indefinite follow-up for all patients [9].

The prognosis of SFTs remains yet to be fully elucidated since follow-up data of the few reported cases are limited; however, it is believed that these tumors generally pursue a slow, indolent, and non aggressive course. Recurrence, malignant transformation, or cerebrospinal fluid dissemination has also been described, though seemingly not as frequently. In these cases, meticulous and complete resection, rather than histological grading, is believed to be the most important prognostic factor and may preclude downstream malignant behavior [10]. Histological features such as necrosis, hypercellularity and mitoses did not affect the rate of recurrence. A high proliferation index should be considered as a prognostic parameter. Michele et al advocated inclusion of high Ki-67 (i.e. > 5%) as an adverse prognostic parameter in assessing the prognosis of SFT of the CNS [11,12].

Although an infrequently encountered tumor entity, it is important to raise awareness of SFTs in neurosurgeons and neuro-oncologists alike as a distinct entity in the differential diagnosis of CNS tumors. Even though SFT is considered benign, it should be followed up regularly due to a possible recurrence [8,11].

## 4 Conclusion

To conclude, SFTs of the CNS are rare entities that are challenging to manage. For its rarity and resemblance to other more common brain tumors, they are often poorly recognized and remain a diagnostic challenge. Careful radiological and histopathological examination is mandatory to arrive at an accurate diagnosis. On immunohistochemical staining, they are characterized by the positive expression of STAT 6, CD34 and Bcl-2. Surgery is the treatment of choice, and some reports recommend adjuvant chemoradiotherapy. Since the potential for malignant transformation exists, we recommend diligent long-term follow-up including regular imaging surveillance.

## Conflicts of interest

We have no conflict of interest.

## Source of funding

Nil.

## Ethical approval

Ethically approved.

## Consent

Written informed consent was obtained from the patient for publication of this case report and accompanying images. A copy of the written consent is available for review by the Editor-in-chief of this journal on request.

## Author contribution

Study concept: Dr. Sant Prakash Kataria, DR. Sanjay Kumar.

Writing the paper: Dr. Namita Bhutani, Dr. Ishwar Singh.

Data interpretation: Dr. Rajeev Sen, Dr. Gajender Singh.

## Registration of research studies

Not applicable.

## Guarantor

Dr. Namita Bhutani.

## Provenance and peer review

Not commissioned, externally peer reviewed.
